# CNN-LSTM Hybrid Model to Promote Signal Processing of Ultrasonic Guided Lamb Waves for Damage Detection in Metallic Pipelines

**DOI:** 10.3390/s23167059

**Published:** 2023-08-09

**Authors:** Li Shang, Zi Zhang, Fujian Tang, Qi Cao, Hong Pan, Zhibin Lin

**Affiliations:** 1 Department of Civil and Environmental Engineering, North Dakota State University, Fargo, ND 58018, USA; li.shang@ndsu.edu (L.S.); zi.zhang@ndsu.edu (Z.Z.); 2 School of Civil Engineering, Dalian University of Technology, Dalian 116024, China; ftang@dlut.edu.cn (F.T.); qcao@dlut.edu.cn (Q.C.)

**Keywords:** CNN-LSTM hybrid model, Lamb wave, data-driven approach, damage identification, structural health monitoring, machine learning

## Abstract

The ultrasonic guided lamb wave approach is an effective non-destructive testing (NDT) method used for detecting localized mechanical damage, corrosion, and welding defects in metallic pipelines. The signal processing of guided waves is often challenging due to the complexity of the operational conditions and environment in the pipelines. Machine learning approaches in recent years, including convolutional neural networks (CNN) and long short-term memory (LSTM), have exhibited their advantages to overcome these challenges for the signal processing and data classification of complex systems, thus showing great potential for damage detection in critical oil/gas pipeline structures. In this study, a CNN-LSTM hybrid model was utilized for decoding ultrasonic guided waves for damage detection in metallic pipelines, and twenty-nine features were extracted as input to classify different types of defects in metallic pipes. The prediction capacity of the CNN-LSTM model was assessed by comparing it to those of CNN and LSTM. The results demonstrated that the CNN-LSTM hybrid model exhibited much higher accuracy, reaching 94.8%, as compared to CNN and LSTM. Interestingly, the results also revealed that predetermined features, including the time, frequency, and time–frequency domains, could significantly improve the robustness of deep learning approaches, even though deep learning approaches are often believed to include automated feature extraction, without hand-crafted steps as in shallow learning. Furthermore, the CNN-LSTM model displayed higher performance when the noise level was relatively low (e.g., SNR = 9 or higher), as compared to the other two models, but its prediction dropped gradually with the increase of the noise.

## 1. Introduction

Onshore transmission/distribution oil/gas pipelines are major energy systems to transport and deliver energy to power communities and other end users. These pipeline structures are vulnerable to mechanical damage, corrosion, and other threats when subjected to aging and different stressors. Different NDT-based sensors and inline inspection tools, including ultrasonic guided lamb waves, have been used to monitor, detect, and locate potential defects in pipelines. 

Besides the physics-based signal processing, data-driven approaches have been accepted in the recent decade for data processing, including the use of artificial neural networks (ANNs) [1,2,3] and deep learning methods (DLs) [4,5,6]. Waveform-based deep neural networks have become a necessary part of many pattern recognition systems [7,8,9,10,11]. These deep neural networks directly take raw signals as input, such as in infrastructure condition assessment [12], stress level identification [13], structure damage identification [14,15], structure health monitoring [16,17,18,19], structure damage diagnosis [20,21,22], and structure damage detection [23,24,25]. 

For feature extraction in ultrasonic signal processing, Pittner and Kamarthi [26] developed a method to automatically sort the wavelet coefficients matrix into an important frequency range and a less important frequency range and used the Euclidean norms to calculate the features of a process wavelet signal [26]. This method was successfully applied to the diagnosis of pulmonary diseases [26]. Shi et al. concluded that the multi-stable stochastic resonance (SR) method showed much better performance and capability in signal processing than the classical bistable SR method [27]. It could improve the output signal-to-noise ratio and the detection effect and also detect lower weak signal-to-noise-ratio signals [27]. Shi et al. presented a method of empirical mode decomposition with cascaded multi-stable stochastic resonance system (CMSRS) denoising. They found that this method could effectively help denoise high-frequency signals, improve the energy of low-frequency signals, and identify fault characteristic signals [27]. Zhao et al. [28] proposed a piecewise tri-stable stochastic resonance (PTSR) method to extract signal fault features and compared the fault characteristics of the extracted signals with those obtained with the standard tri-stable SR method. The result showed that the PTSR method had better signal processing performance than the STSR method [28]. Despite that, the applicability of the CNN-LSTM hybrid model has not been investigated in the fault ultrasonic signal classification area. In addition, the feature parameters used to express the features of time-series data have not been used with the ultrasonic signals of damaged pipelines. 

Therefore, this study aimed to fill the knowledge gaps in how to achieve efficient and accurate classification of different kinds of corrosion damage in pipelines by utilizing the multi-feature extraction capability of the hybrid deep learning model and constructing reasonable datasets to improve the accuracy of different models. In this study, the dataset used for model training was compiled from field tests, and the classification results of the proposed CNN-LSTM hybrid model were compared with those of some benchmark models, like the CNN and LSTM models, to verify its advantages. Furthermore, the influence of the signal noise on the classification accuracy was specifically determined.

This article is organized as follows: firstly, the method of wavelet threshold denoising was used to denoise the raw ultrasonic signal series. Secondly, twenty-nine feature parameters were extracted as input data for training different machine learning networks. Next, data reduction was used to reduce the dimensionality of the twenty-nine feature parameters series, and the reduced data were input to the CNN-LSTM hybrid model to verify the classification accuracy. Finally, the impact of noise interference on the effectiveness of the CNN-LSTM model was further evaluated.

## 2. Framework of Machine Learning-Enriched CNN-LSTM Method for Damage Detection

**Figure 1:**
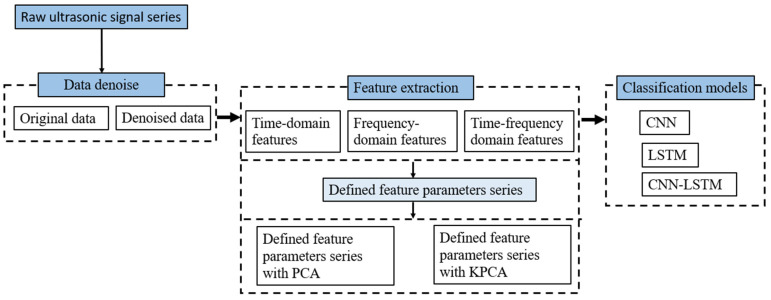
Framework of the machine learning-enriched method for damage detection.

Figure 1 shows the flowchart of the methodology. The sequences of monitoring data were collected to build the dataset. The dataset included different kinds of pipeline defects. Second, the CNN-LSTM model was established and verified as compared to CNN and LSTM, individually, and further studies were conducted to reveal the influence of the different features and the data reduction process on the prediction accuracy.

### 2.1. CNN-LSTM Hybrid Model

**Figure 2:**
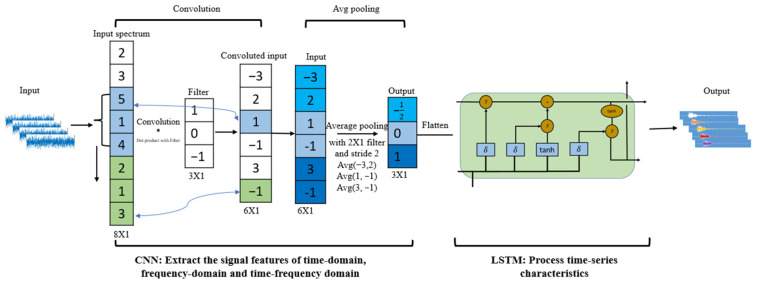
Flow chart of the CNN-LSTM hybrid model.

A CNN-LSTM hybrid model is proposed, and the structure and the data processing flow of the model are shown in Figure 2, which is based on Zhang’s research [29]. The purpose of the CNN layer is to extract the signal features of the time domain, frequency domain, and time–frequency domain from the monitoring data. The obtained features were then put into a two-dimensional array and used as the input for the LSTM layer to analyze the time series features. The mechanism of feature extraction of CNN and feature processing of LSTM is shown in the following sections. In the CNN, LSTM, and CNN-LSTM models, batch normalization layers were constructed to normalize the outputs of each layer, which lowered the overfitting risks and increased the stability of the optimization process. The function of the batch normalization layers has also been demonstrated by Xu and Andhale’s research [30,31].

The convolutional neural network (CNN) is a popular deep learning algorithm, and its purpose is to process data in different dimensions [32]. The convolutional layer and the max-pooling layer are the two main layers in the CNN structure, as shown in Figure 1.

The convolutional layer is designed to perform convolution and activation operations on the input data and produce feature maps [29]. The mathematic procedure of convolution in layer l is presented in [33], as shown below:

(1) $$C_{j}=f\left(\sum_{mi\;E\;M}mi\times kj+bj\right)$$

where mi is the data input to the convolutional layer, kj is the convolutional kernel, and bj is the bias; f(·) represents the activation function.

The average pooling layer follows the convolutional layer and helps to reduce the feature map resolution and decrease the network computation time. The arithmetical formula of the pooling operation in layer l is shown in [33]:

(2) $$Sj=\beta j\;down\left(cj\right)+bk$$

where *down* (·) represents the average pooling method.

The input dataset for the CNN-LSTM hybrid model is collected from time series data. For the CNN structure, the input layer works with the input data, the convolutional layer extracts data features with kernel functions, and the average pooling layer is to reduce the amount of data from the convolutional layer, reducing overfitting [34]. Finally, the data are flattened into the LSTM layer.

LSTM is a long short-term memory network and is an effective tool to deal with sequence and time series data for classification and regression problems [35]. The LSTM network defines three layers. The sequence input layer and the LSTM layer are the two most important structures of an LSTM network. The purpose of the sequence input layer is to input the time series data for the LSTM network. The purpose of the LSTM layer is to memorize long-term dependencies between time steps of sequence data [35]. The last layer is used to output the information of pattern recognition.

**Figure 3:**
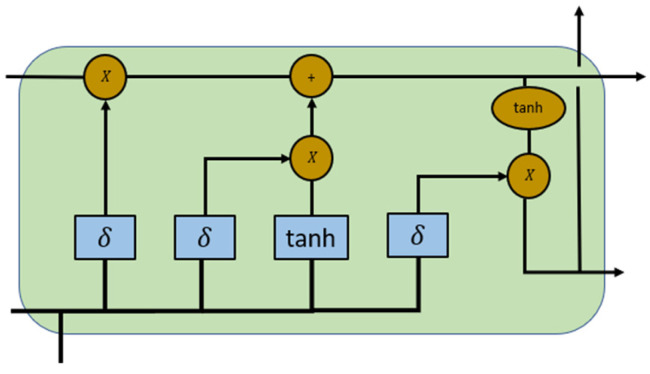
The LSTM structure of a cell [35].

There are four components, i.e., input gate (*i*), forget gate (*f*), cell candidate (*g*), and output gate (*o*), used to control the cell state and the hidden state of the layer [36]. Figure 3 shows the LSTM structure, which was drawn based on Chevalier’s research [35], illustrating the flow of data at the time step *t*.

The input gate ($i_{t}$) and the forget gate ($f_{t}$) are defined to control the cell state update and reset (forget), respectively, while the cell candidate ($g_{t}$) and the output gate (*o*) denote the added information to the cell state and control the cell state added to the hidden state, respectively, as shown in [36]:

(3) $$i_{t}=\sigma_{g}\left(W_{i}x_{t}+R_{i}h_{t-1}+b_{i}\right)$$

(4) $$f_{t}=\sigma_{g}\left(W_{f}x_{t}+R_{f}h_{t-1}+b_{f}\right)$$

(5) $$g_{t}=\sigma_{c}\left(W_{g}x_{t}+R_{g}h_{t-1}+b_{g}\right)$$

(6) $$o_{t}=\sigma_{g}\left(W_{o}x_{t}+R_{o}h_{t-1}+b_{o}\right)$$

where *t* is the time step, $\sigma_{g}$ is the gate activation function, the matrices *W*, *R*, and *b* are concatenations of the input weights and recurrent weights and the bias of each component, respectively.

$c_{t}$ is the cell state at the time step *t* and can be defined as in [36]:

(7) $$c_{t}=f_{t}⨀c_{t-1}+i_{t}⨀g_{t}$$

where ⨀ is the Hadamard product.

$h_{t}$ is hidden state at the time step *t* and can be defined as in [36]:

(8) $$h_{t-1}=o_{t}⨀\sigma_{c}\left(c_{t}\right)$$

where $\sigma_{c}$ is the state activation function.

Figure 3 shows that LSTM can deal with continuous and highly correlated time series data [29]. During the corrosion process of damaged pipeline systems, the current corrosion monitoring data have a closely connection with damage data of the previous days, and the series of corrosion monitoring data is highly time-dependent [29]. As a result, LSTM can be used to deal with time series information from CNN networks and process the processed data to the layers to classify the different kinds of damage in pipelines.

### 2.2. Features Extraction

#### 2.2.1. Definition of Features

In this study, ten dimensional time-domain characteristic indicators, six dimensionless time-domain characteristic indicators, and thirteen frequency-domain characteristic indicators were selected to characterize the fault characteristics in different damaged pipelines, as shown in Table 1 and Table 2. Here, sixteen time-domain features and thirteen frequency-domain features were chosen. These indicators were chosen based on Chen’s research [37].

The dimensional indicators become much bigger with the development of defects and also change with changes in working conditions [37]. Dimensionless indicators depend on the probability density function. The two types of indicators together are expected to better reflect the trend of pipeline defects. Therefore, this study used these indicators as a time-domain characteristic index. These indicators are usually applied to reflect the fault trend of space rolling bearings [37].

In Table 2, $s\left(k\right)$ is the spectrum of signal *x*(*n*), *k* = 1, 2, 3…, *K*, *K* is the number of spectral lines, and $f_{k}$ is the frequency value of the *k*th spectral line. The characteristic parameter $p_{1}$ reflects the vibration energy in the frequency domain, $p_{2}$, $p_{3}$, $p_{4}$, $p_{6}$ and $p_{10}–p_{13}$ reflect the degree of dispersion or concentration of the spectrum, $p_{5}$, $p_{7}$, $p_{8}$ and $p_{9}$ reflect the change of the position of the main frequency [37].

#### 2.2.2. Data Dimension Reduction

The number of features in the time domain, frequency domain, and time–frequency domain was too large. In order to select several main characteristics to express the fault features of damaged pipelines, two different methods of data dimension reduction were tested, i.e., the PCA method and the K-PCA method.

##### Principal Component Analysis (PCA)

PCA could be defined as follows:

Step 1: standardization.

(9) $$z=\frac{value-mean}{standard\;deviation}$$

After standardization, all the variables are transferred into the range of [0, 1], to reduce the deviation of the principal components.

Step 2: Covariance matrix calculation.

(10) $$C=\frac{1}{l}\sum_{t=1}^{l}X_{t}{X_{t}}^{T}$$

For $X_{t},\sum_{t=1}^{l}X_{t}=0$, $X_{t}={\left(X_{t}\left(1\right),\;X_{t}\left(2\right),\ldots ,X_{t}(m)\right)}^{T}$, *t* = 1, 2, 3,…, *l*, *m* is the dimension and *m* < l.

Step 3: Calculate the eigenvectors and eigenvalues of the covariance matrix to identify the principal components.

(11) $$\lambda_{i}\mu_{i}=C\mu_{i}$$

where $\lambda_{i}$ is one of the eigenvalues of *C*, $\mu_{i}$ is the corresponding eigenvector, *i* = 1, 2, 3,…, *m*.

Step 4: the principal components of $s_{t}$ can be calculated as the orthogonal transformations of $X_{t}$ based on the corresponding eigenvector $\mu_{i}$.

(12) $$s_{t}\left(i\right)={\mu_{i}}^{T}X_{t}$$

##### Kernel Principal Component Analysis (KPCA)

KPCA allows performing nonlinear transformations and achieve a nonlinear analysis from linear PCA on the samples using the kernel method.

(13) $$\lambda_{i}\alpha_{i}=K\alpha_{i}$$

where *K* is the kernel matrix, $K={\left\{K_{ij}\right\}}_{n\times n}$, $K_{ij}=k(x_{i},x_{j})$, $x_{i}$, and $x_{j}$ are samples in the original space.

(14) $$Z^{l}\left(x\right)=\sum_{i=1}^{n}{\alpha_{i}}^{l}k\left(x_{i},x_{j}\right)$$

(15) $$Y={\left[Z^{1}\left(x\right),\;Z^{2}\left(x\right),\;Z^{3}\left(x\right),\ldots ,Z^{m}\left(x\right)\right]}^{T}$$

where $\alpha^{l}$ is the *l*th eigenvector. In the new space, the coordinates of the sample *x* on the first m nonlinear principal components constitute the sample *Y*. KPCA has the same properties as PCA, and KPCA can extract a greater number of principal components than PCA.

### 2.3. Evaluation of the Model Performance

#### 2.3.1. Confusion Matrix and Accuracy as Performance Indicators

A confusion matrix is a popular tool applied to classification problems, including binary classification and multiclass classification problems [38]. Table 3 is an example of a confusion matrix for binary classification [38].

The counts of predicted and actual values are calculated from confusion matrices. The output “*TN*” indicates True Negative, which is the number of negative examples classified accurately [38]. “*TP*” indicates True Positive, which shows the number of positive examples classified accurately [38]. “*FP*” stands for False Positive, which means the number of actual negative examples classified as positive [38]. “*FN*” is False Negative, which is the number of actual positive examples classified as negative [38]. The accuracy of a confusion matrix model is calculated using the formula below [38].

(16) $$Accuracy=\frac{TN+TP}{TN+FP+FN+TP}$$

#### 2.3.2. ROC Curve as Another Performance Indicator

Receiver operating characteristic (ROC) curves are produced by comparing the true positive rate to the false positive rate, depending on various thresholds, and are used as an evaluation tool in machine learning [17,21]. The area under the ROC curve (AUC) indicates the level of separability and ranges from 0 to 1. A better model performance is associated with a higher AUC. When a model has an accuracy of 100%, the AUC equals one.

## 3. Case Study

### 3.1. Ultrasonic Guided Waves Collected from Embedded Damaged Pipes

**Figure 4:**
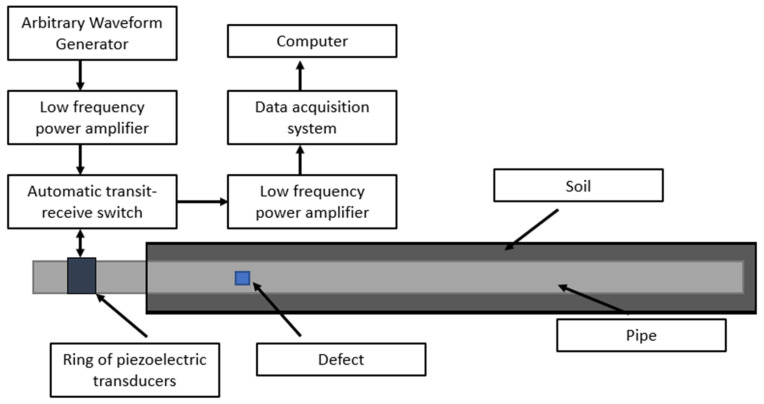
Experimental principle.

**Figure 5:**
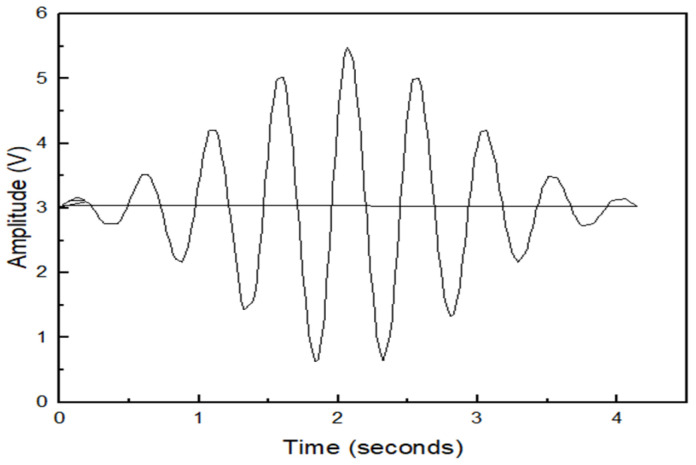
Tone burst signal.

Figure 4 shows the experiment principle of the ultrasonic testing system. Torsional guided waves were excited using piezoelectric transducers by manipulating their orientation, as reported in the literature [39]. A total of nine piezoelectrical transduces were arranged axially in a ring to build the test system. Tone burst signals [31] were used to excite the transducers, and the low bandwidth nature of these signals made the generation of torsional mode much easier, as shown in Figure 5. The waveform generator was the 33220A 20 MHz Function/Arbitrary Waveform Generator. The parameters of Arbitrary Waveform Generator were as follows. The waveform type was the default arbitrary waveform with frequency of 40 Khz, amplitude of 10 vpp, and waveform production period of 3 s. In order to better read the waveform data, the Noesis 7.0 software was used to read the original waveform data which will be fed into the neural networks.

**Figure 6:**
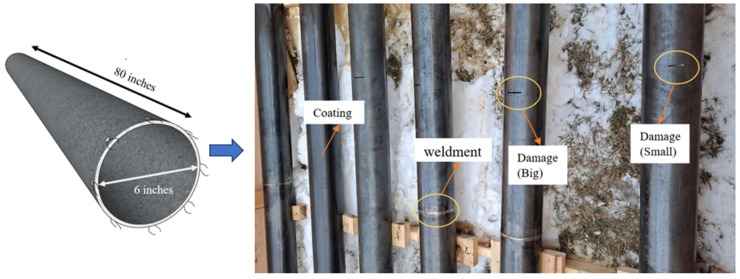
Test samples.

**Figure 7:**
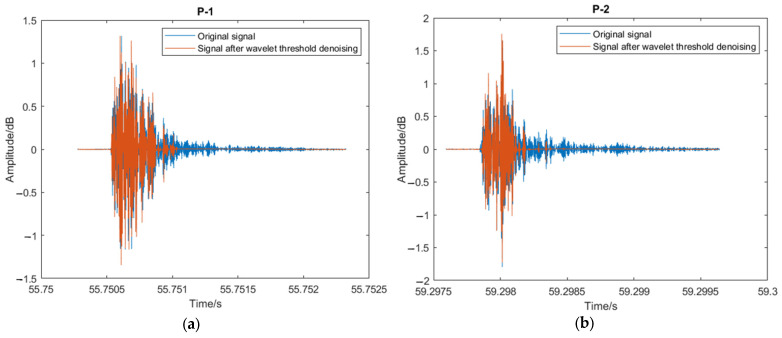

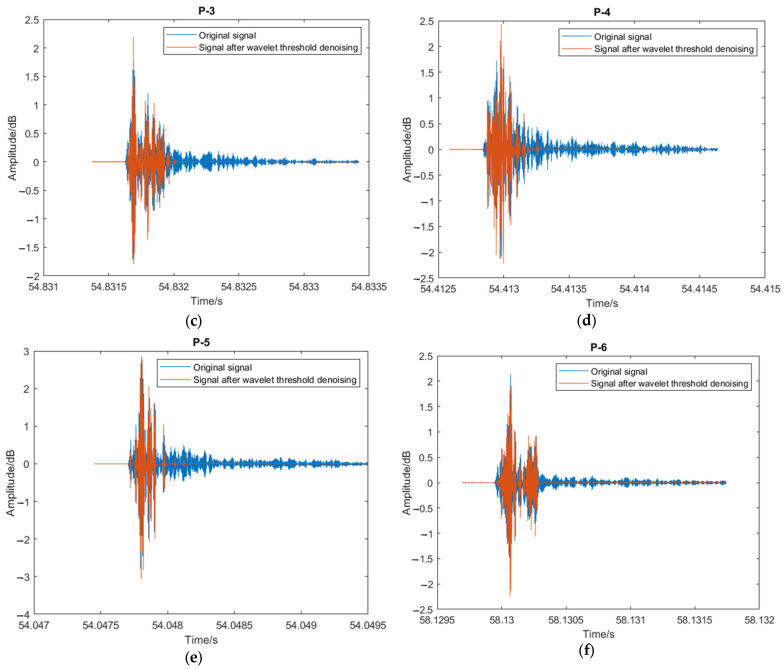
Original signal and signal after wavelet threshold denoising.

Figure 6 shows the experimental setup. Six kinds of pipelines were designed for field testing. The pipeline samples had a 6-inch diameter and an 80-inch length. A total number of 336 groups of samples were collected for each pipeline state, of which 240 were randomly selected as training samples, and the remaining 96 were used as test samples. Each set of samples contained 3000 sampling points. This dataset consisted of six classes, and each class represented different kinds of damaged pipes (Table 4), as follows: (a) P-1, the pipe had a small notch located at 1/3 L away from the left side, (b) P-2, the pipe had a big notch located at 1/3 L away from the left side and a weldment at 2/3 L away from the left side, (c) P-3, the pipe had a small notch at 1/3 L and a weldment at 2/3 L away from the left side, (d) P-4, the pipe had a big notch located at 1/3 L away from the left side, (e) P-5, the pipe had an epoxy coating without damage, and (f) P-6, the pipe had an epoxy coating with a weldment at 2/3 L away from the left side. The reason for this pairing was to detect the steps in the corrosion of different kinds of pipes. The specific description of the data set is shown in Table 4. The time-domain waveforms corresponding to the various pipeline states are shown in Figure 7. It is clear to see that there has huge difference between original signals and noised signals. 

### 3.2. Data Denoiinge Using Wavelet Threshold Denoising

Wavelet threshold denoising can be realized through the following steps, based on [40].

Step 1: Discrete wavelet decomposition of signal with noise. According to the characteristics of the signal with noise, the appropriate wavelet base and the number of decomposition layers are selected to perform discrete wavelet transform, and the wavelet coefficients $d_{j,k}$ of each layer are acquired.

The one-dimensional non-stationary signal model is as follows [41]:

(17) $$x\left(t\right)=f\left(t\right)+\varepsilon \left(t\right)$$

where $x\left(t\right)$ is the original signal with noise, *f*(*t*) is the original signal without noise, ε(t) is the white Gaussian noise signal.

(18a) $$\int x\left(t\right)\psi_{j,k}\left(t\right)dt=\int f\left(t\right)\psi_{j,k}\left(t\right)dt+\int \varepsilon \left(t\right)\psi_{j,k}\left(t\right)dt$$

(18b) $$d_{j,k}=u_{j,k}+e_{j,k}$$

where $\psi_{j,k}(t)$ is the discrete wavelet basis function, and $d_{j,k}$ is the wavelet coefficient of each layer after the wavelet transformation of the signal with noise *x*(*t*); $u_{j,k}$ is the wavelet transformation coefficient of the original signal *f*(*t*); $e_{j,k}$ is the wavelet transformation coefficient of the white Gaussian noise signal *ε*(*t*).

Step 2: Threshold quantization processing. The threshold λ and the threshold function are used to process the wavelet coefficients $d_{j,k}$ to obtain the processed wavelet coefficients $d_{j,k}^{\prime }$ of each layer.

Step 3: Wavelet coefficient reconstruction. The processed wavelet coefficients $d_{j,k}^{\prime }$ and the approximate coefficients of the jth layer are reconstructed to obtain the denoised signal *x*’(*t*).

Figure 7 shows the original signal and the signal after wavelet threshold denoising. Signal denoising enhances the signal-to-noise ratio by eliminating interferences that do not supply relevant information and reduce the predicted accuracy of machine learning models [42].

## 4. Results and Discussion

### 4.1. Classification Performance of CNN, LSTM, and the CNN-LSTM Model with Twenty-Nine Feature Parameter Series

In order to demonstrate the effectiveness of the established CNN-LSTM model for data classification, this study build the CNN and LSTM models as benchmark models and used the twenty-nine feature parameters series in Table 1 and Table 2 as the dataset for model training. This study used padding to prevent information loss when a CNN was utilized for feature extraction [29]. The classification performance of the CNN, LSTM, and CNN-LSTM models was compared. The classification accuracy was evaluated by confusion matrix and according to the expression of accuracy in Equation (16).

**Figure 8:**
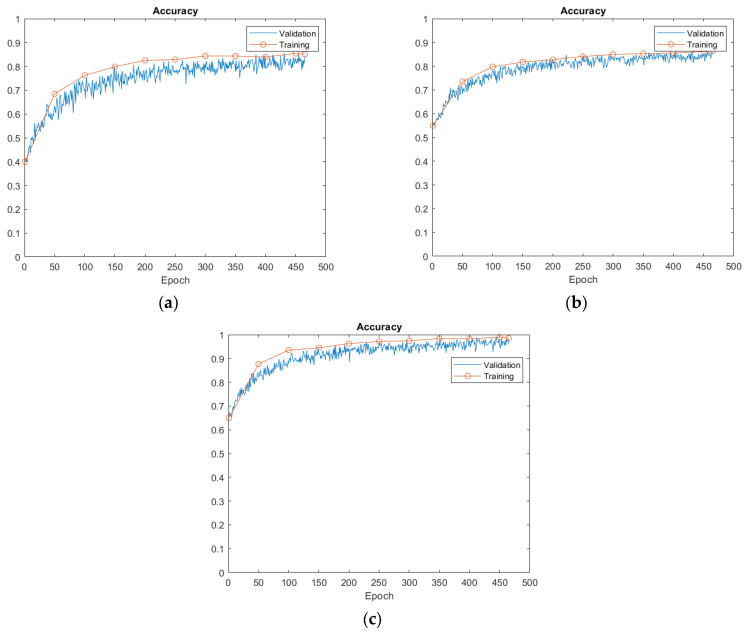
Accuracy of training and validation for the three models. (**a**) CNN; (**b**) LSTM; (**c**) CNN-LSTM.

A CNN-LSTM hybrid model was established as discussed in Section 2.1 and as shown in Table 5, and the hybrid model was utilized to concurrently extract the temporal features and analyze the time series features of the dataset. Figure 8 shows the training progress for both the training and the validation sets of the three models over 300 epochs. The CNN-LSTM model achieved better performance than the CNN and LSTM models at the very beginning. For instance, the accuracy of the CNN-LSTM model on both the training set and the test set started from 65% with epoch = 0, while the accuracy of the CNN and LSTM models on both the training set and the test set was 40% and 55%, respectively.

As the number of epochs increased, the accuracy on the training set and test set of the three models also showed a rising trend. Furthermore, it is clear that the training accuracy was much higher than the validation accuracy for the three models. When the number of epochs reached 300, the model training accuracy and validation accuracy reached the highest values, i.e., 94.8% for the CNN-LSTM model, 86.5% for the LSTM model, and 85.4% for the CNN model, as shown in Table 5. The classification accuracy on both the training set and the test set was stable at about the highest value at the same time, which means that the model was capable of adjusting to the training set.

**Figure 9:**
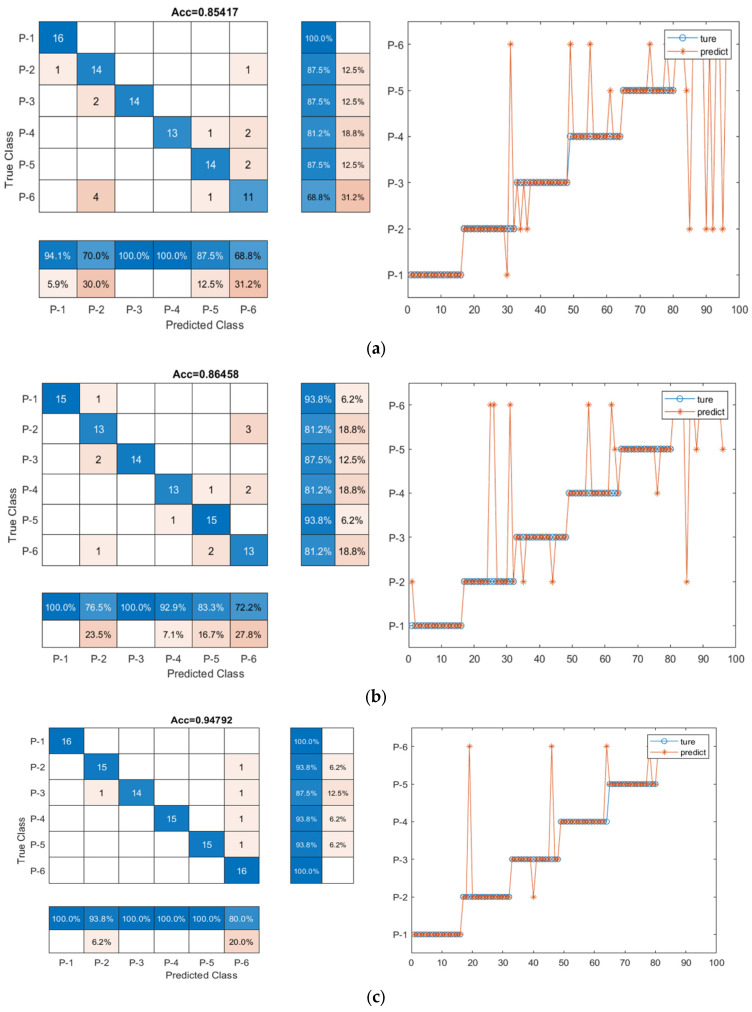
The confusion matrix for the three models. (**a**) CNN; (**b**) LSTM; (**c**) CNN-LSTM.

Table 5 shows the test results of the three models, and a total number of twenty-nine feature parameters series were used as input. Clearly, the CNN-LSTM hybrid model had a much higher accuracy (94.8%), as compared to the CNN and the LSTM models, with accuracy of 85.4% and 86.5%, respectively. To provide a more intuitive comparison, the confusion matrix predicted for each model is shown in Figure 9. CNN-LSTM displayed five signals out of ninety-six testing signal samples that were mistakenly categorized into other groups, while CNN and LSTM included fourteen and thirteen out of ninety-six signals incorrectly grouped, respectively, suggesting that the CNN-LSTM hybrid network could have higher capability for data classification. Even though the CNN and LSTM models showed identical accuracy, the slightly higher accuracy of the LSTM model could be partially due to the fact that the LSTM structure is specifically proposed for dealing with time series data, as in this study, thus leading to slightly better results, which was also confirmed in the other experiments, as reported below. To evaluate the training efficiency of the three models (CNN, LSTM, and CNN-LSTM models), the training time was calculated and compared, as shown in Table 5. The CNN model can be computationally demanding, especially for large structures or deep layers, and its training time was the least, 30 s, while the LSTM model can take longer to train compared to the CNN model, particularly for long input sequences. The CNN-LSTM model took the longest time (45 s) for training, because the CNN-LSTM model integrates the complexities of both the CNN model and the LSTM model, which makes them more computationally intensive.

### 4.2. Classification Performance of the CNN-LSTM Model with Denoised Data

**Figure 10:**
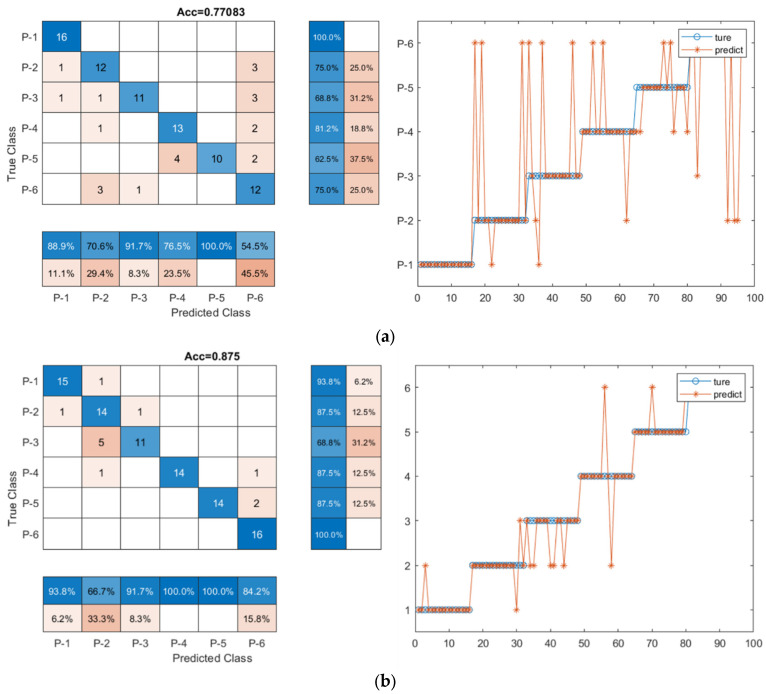

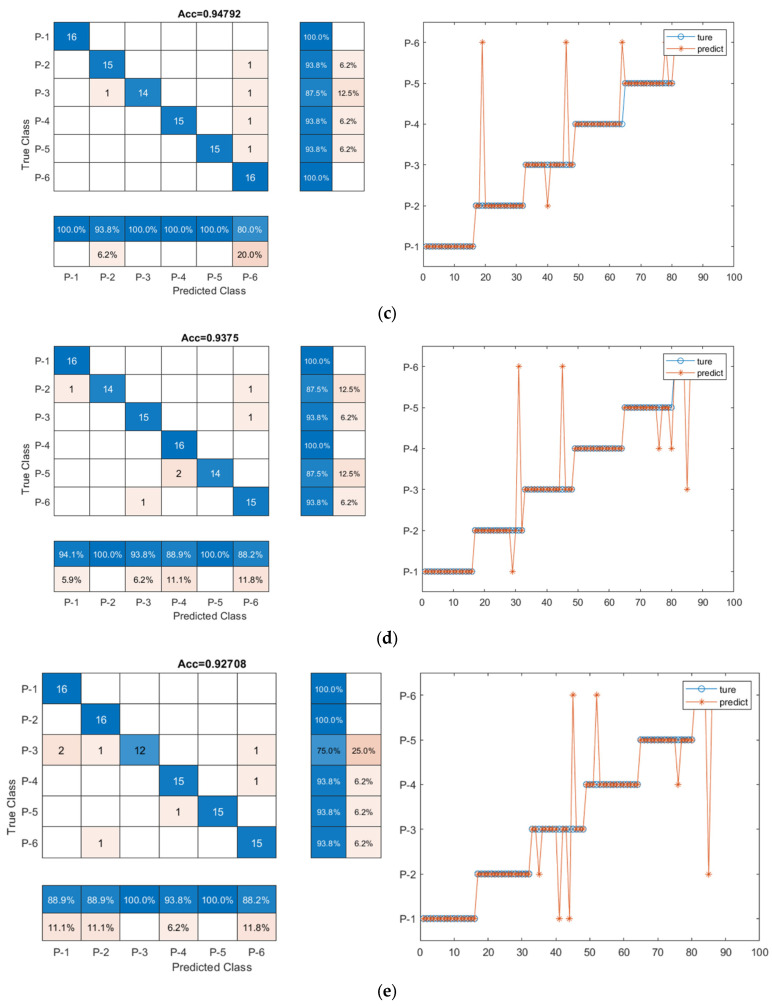
The confusion matrix of the CNN-LSTM model with different kinds of input data. (**a**) Original data; (**b**) Denoised data; (**c**) Twenty-nine feature parameter series; (**d**) Twenty-nine feature parameter series with PCA; (**e**) Twenty-nine feature parameter series with KPCA.

**Figure 11:**
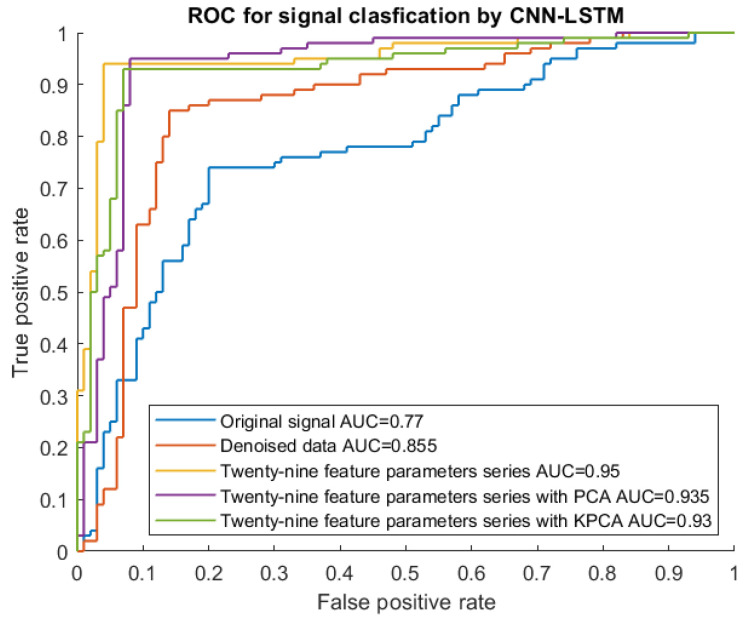
ROC curve for the CNN-LSTM hybrid model with different input data.

To quantitatively study how denoised data improved the classification accuracy of the deep learning models, the CNN-LSTM model was trained using the dataset without denoise (original signal dataset) and with denoise. The denoised data set was clearly different from the original data, as shown in Figure 7. The training accuracy, confusion matrix, and ROC curve were used as indicators for the comparison, as shown in Table 6 and Figure 10 and Figure 11. When considering the denoise, the accuracy of the CNN-LSTM model was 87.5%, improving by 11% compared to the accuracy (77.1%) of the CNN-LSTM model with the original data. As illustrated in Figure 10, seven signal samples out of ninety-six testing signal samples were incorrectly placed into other groups using the denoised data model, and twenty-two signal samples out of ninety-six testing signal samples were mistakenly grouped into other groups using the original data model. Similarly, as shown in Figure 11, the AUC of the CNN-LSTM model with denoised data was 0.855, which was also larger than that of the CNN-LSTM model with original data (0.770). This demonstrated that when denoise was considered in the dataset, the classification accuracy of the CNN-LSTM hybrid model could be improved.

### 4.3. Classification Performance of the CNN-LSTM Model with Predetermined Features

To analyze the effectiveness of the twenty-nine feature parameters, the CNN-LSTM model was trained using the dataset with and without the twenty-nine feature parameters. As shown in Table 6 and Figure 10, the classification accuracy of the CNN-LSTM model with the twenty-nine feature parameter series improved by 22.97% and 8.33%, respectively when compared to that achieved with the original input data (77.083%) and the denoised input data (87.500%). The AUC of the CNN-LSTM model with twenty-nine feature parameters was the highest (0.950), close to 1, as shown in Figure 11. The result is meaningful and indicated that feature extraction can help improve the training accuracy and performance of the CNN-LSTM hybrid model and the twenty-nine feature parameter series can be used as an indicator of fault signal features to detect pipeline damage.

### 4.4. Classification Performance of the CNN-LSTM Model with Data Dimension Reduction

To further improve the classification accuracy of the CNN-LSTM model, the feature dimension can be optimized. In this study, PCA and KPCA were applied to decrease the feature dimension of the twenty-nine feature parameters, and the CNN-LSTM model with and without reduction of feature dimension was trained. As shown in Table 6 and Figure 10, when the twenty-nine feature parameter series with PCA was used as input data of the CNN-LSTM hybrid network, the classification accuracy was 93.8%. When the twenty-nine feature parameters with KPCA were used as input data of the CNN-LSTM hybrid network, the classification accuracy was 92.7%. The classification accuracy was reduced by 1% and 2%, for the model with the twenty-nine feature parameter series with PCA and the model with the twenty-nine feature parameter series with KPCA, respectively, compared with that pf the network with the twenty-nine feature parameter series input (94.8%). For the ROC curve in Figure 11, the AUC values are 0.935 and 0.930 for the model with the twenty-nine feature parameters with PCA and the model with twenty-nine feature parameters with KPCA, respectively, which was also lower than the AUC of the CNN-LSTM model with twenty-nine feature parameters (0.950). The result indicated that the reduction of the data dimension did not effectively promote the classification accuracy but might reduce the classification accuracy to some extent.

## 5. Further Discussion of the Effectiveness of the Hybrid Model under Noise Interference

To evaluate the performance and robustness of the signal processing and model training in the previous study, the noise interference on feature extraction and model training were studied. Specifically, the white Gaussian noise was added to original signal to simulate the real situation with noise; the noise levels were from 3 dB to 15 dB.

### 5.1. Introduction of White Gaussian Noise into the Signals

**Figure 12:**
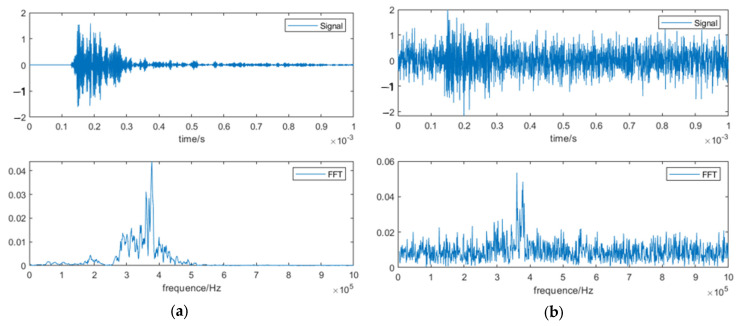

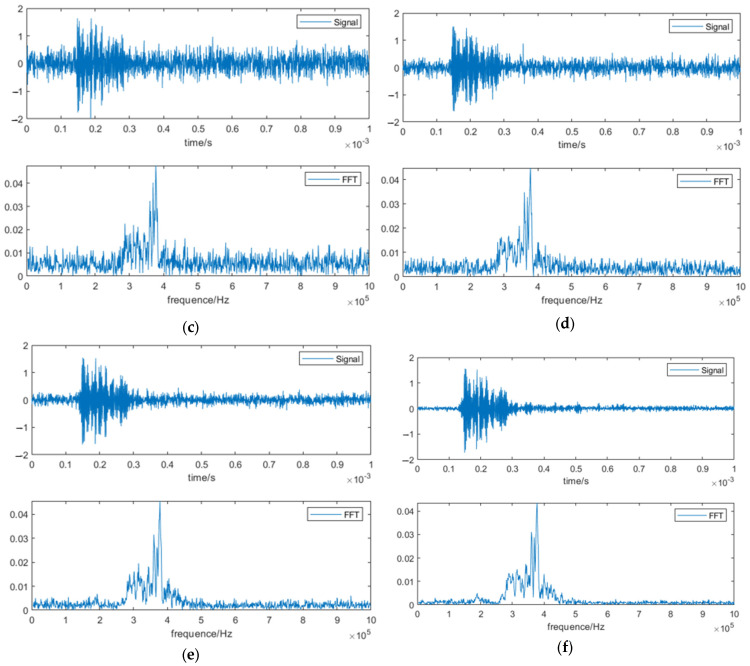
The noised signals at different noise levels. (**a**) Original signal; (**b**) SNR = 3 dB; (**c**) SNR = 6 dB; (**d**) SNR = 9 dB; (**e**) SNR = 12 dB; (**f**) SNR = 15 dB.

To study the robustness of signal processing and model training, the white Gaussian noise was directly added to the original signal data. Taking the signal in P-1 as an example, Figure 12 shows the signals with different noise interference. It is clear that with the increase of the SNR, the signal became increasingly clear. When SNR = 15 dB, the signal was almost the same as the original signal. When SNR = 3 dB, the signal was contaminated by noise, and it was hard to differentiate between noise and signal. The sensitivity of the deep learning algorithm to the uncertainty brought on by noise was also tested by classifying the signals at various noise levels.

### 5.2. Classification Performance of the CNN-LSTM Model with White Gaussian Noise Interference

**Figure 13:**
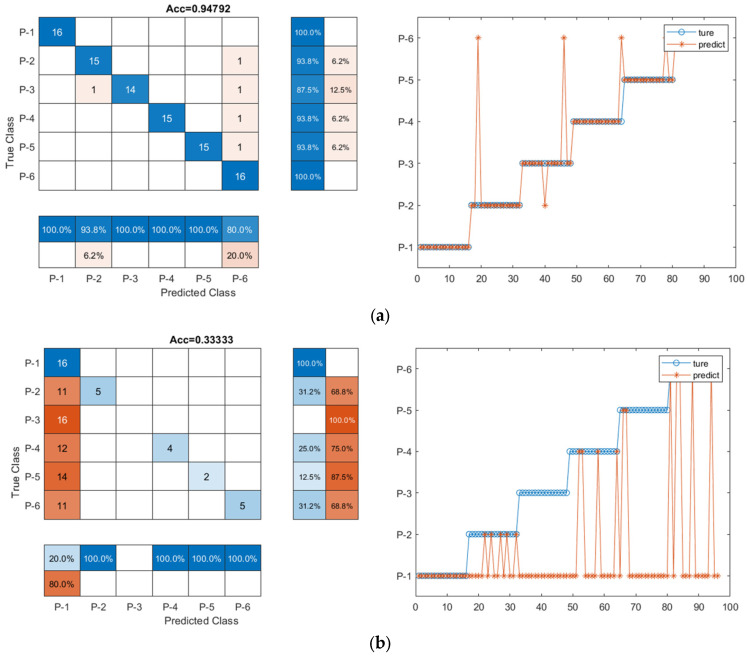

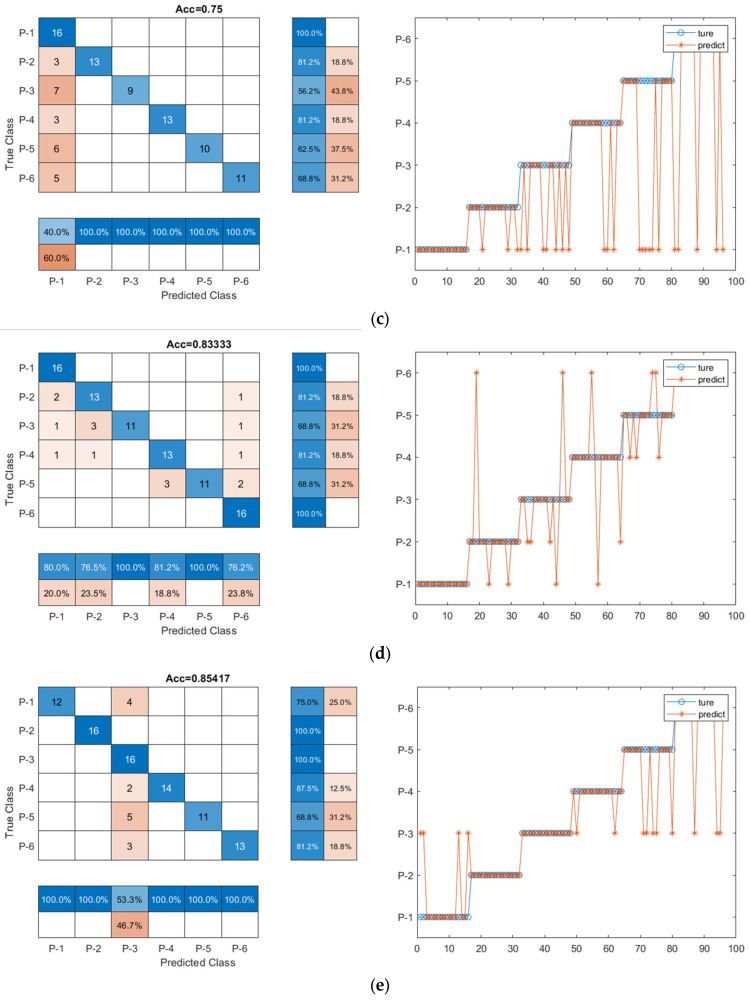

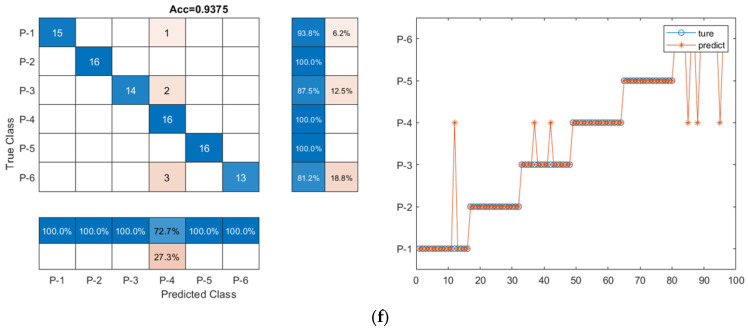
The classification accuracy of the CNN-LSTM model for different SNR. (**a**) Original signal; (**b**) SNR = 3 dB; (**c**) SNR = 6 dB; (**d**) SNR = 9 dB; (**e**) SNR = 12 dB; (**f**) SNR = 15 dB.

To investigate the sensitivity and effectiveness of the model training under noise interference, the CNN-LSTM model was trained with the dataset with and without noise. Different noise levels were considered, as shown in Figure 12. The twenty-nine feature parameters were extracted from the original signal and the noised signal to be used as input of the CNN-LSTM model. The accuracy and confusion matrix of the CNN-LSTM model were compared to evaluate the training performance, and the results are shown in Table 7 and Figure 13. Clearly, the classification accuracy improved with the increase of SNR. A higher SNR indicates a stronger and perceptible signal in comparison to noise, which was consistent with the result. For instance, when SNR = 15 dB, the accuracy of the reconstructed signals was the highest (93.8%), and P-1 and P-2 were completely categorized into the correct groups, but 13% of P-3 and 6% of P-2, P-4, and P-5 were mistakenly placed into wrong groups, as shown in Figure 13. When SNR = 15 dB, there was almost no noise in the signal, as shown in Figure 12, which demonstrated a high accuracy. In contrast, for SNR = 3 dB, the signal was seriously contaminated by the noise, and the accuracy was the lowest (33.3%); the mislabeled data mainly occurred in P-2, P-3, P-4, P-5, and P-6. The misjudgments in these five categories were higher than 69%, which means the features of the signal were hard to extract. With the decrease of the noise level, the data classification accuracy improved. For example, when SNR = 6 dB, 75% of the data could be classified into the correct groups, and the misclassification was mainly in P-2, P-3, P-4, P-5, and P-6, 19%, with 44%, 19%, 38%, and 31% misclassification rate, respectively. When SNR = 9 dB and 12 dB, the misclassification rate decreased. When SNR = 15 dB, the accuracy of the CNN-LSTM model increased by 181. 3%, 25.0%, 20.0%, and 9.8%, respectively when compared with the accuracy of SNR = 3 dB (33.3%), 6 dB (75.0%), 9 dB (83.3%), and 12 dB (85.4%). It means that higher SNR levels could enhance the accuracy by lessening the effect of noise interference and improving the capacity to spot and categorize faults. The results also demonstrated that the signal processing (denoise and feature extraction) and the CNN-LSTM model training were effective under noise interference.

**Figure 14:**
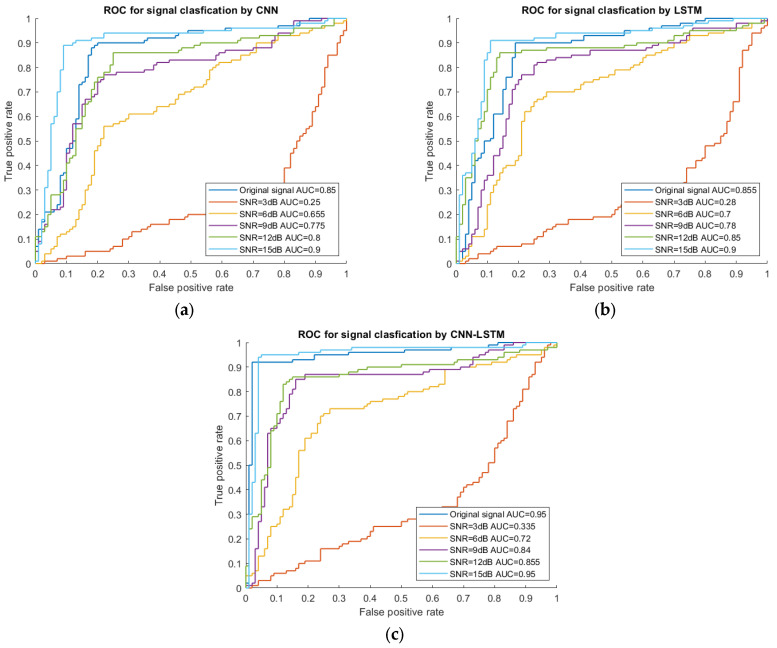
ROC curve for the three models at different noise levels. (**a**) CNN; (**b**) LSTM; (**c**) CNN-LSTM.

The AUC values were calculated to better illustrate the accuracy results, as shown in Table 8 and Figure 14. The AUC values also increased with the decrease of the noise levels, which was consistent with the accuracy results. When SNR = 15 dB, the AUC was 0.950, i.e., close to one. When the noise level was really high (SNR = 3 dB), the AUC value was only 0.335, suggesting that the classification accuracy was unacceptable when the AUC was lower than 0.750, as also shown in the literature [24]. When the noise level decreased to 9 dB and 12 dB, the values of the AUC were 0.840 and 0.855, respectively. The results had the same regularity as the accuracy results; both the accuracy and the AUC values increased with the decrease of the noise levels.

### 5.3. Comparison of the Classification Performance of the CNN, LSTM, and CNN-LSTM Models

This section compares the performance of the three models (CNN, LSTM, and CNN-LSTM) at different levels of white Gaussian noise and used twenty-nine feature parameters as network input. Table 7 and Figure 14 show the comparison results of training accuracy and AUC values of the three models (CNN, LSTM, and CNN-LSTM models). Clearly, the CNN-LSTM hybrid model achieves a better performance than CNN and LSTM at different noise levels due to its complex time-series data processing structure, and this result was also demonstrated in Section 4.1.

For instance, when SNR = 3 dB, the accuracy of the CNN-LSTM model increased by 33% and 16%, respectively, in comparison with those of the CNN (25.0%) and LSTM models (28.8%). The result demonstrated that the CNN-LSTM model had better feature extraction capability than the CNN and LSTM models at a higher noise level, and the LSTM model was much better than the CNN model. With the decrease of the noise level, the difference in training accuracy also decreased for the three models. For instance, when SNR = 15 dB, the performance of the CNN-LSTM model increased by 4% for both the CNN and the LSTM models.

For the AUC values, we found the same trend as for the training accuracy. With the decrease of the noise level, the difference in the AUC values also decreased. For instance, when SNR = 3 dB, the AUC value of the CNN-LSTM model increased by 34% and 20%, respectively, in comparison with those of the CNN and LSTM models, while when SNR = 15 dB, the performance of the CNN-LSTM model increased by 6% with respect to those of both the CNN and the LSTM models. These findings revealed that the CNN-LSTM model still performed better at classifying the data than the CNN and the LSTM models under noise interference.

### 5.4. Detectability of Multiple Defects Using the CNN-LSTM Model

**Figure 15:**
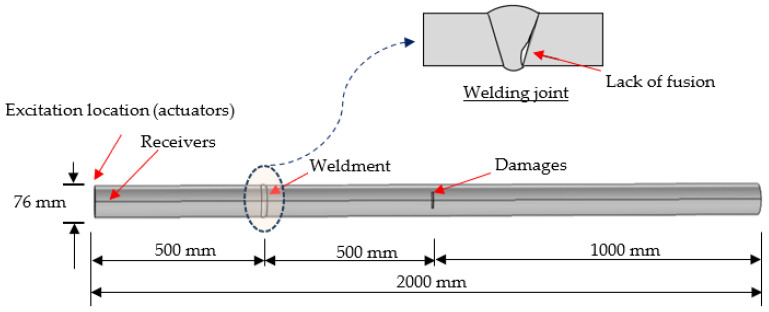
Steel pipe with a welding defect and notch-shaped damage (modified after [16]).

We discussed the robustness of the CNN-LSTM model for damage detection with and without noise interference in Section 4. Note that all cases we used were based on the data classification of a single defect (cracking). As such, we selected one case with multiple defects to demonstrate the effectiveness of the CNN-LSTM approach for damage detection. The case was selected from the authors’ previous work [16], as shown in Figure 15. The pipe dimensions and measurement are identical to those shown in Figure 4 in Section 3, but one crack was located at the middle of the pipe, and the weldment at 1/3 location had a defect due to lack of fusion. More detailed information can be found [16]. The pipe was embedded in 0.5 m deep concrete as an extreme case, leading to a quick signal decay due to high energy loss when the guided waves were transferred in concrete.

**Figure 16:**
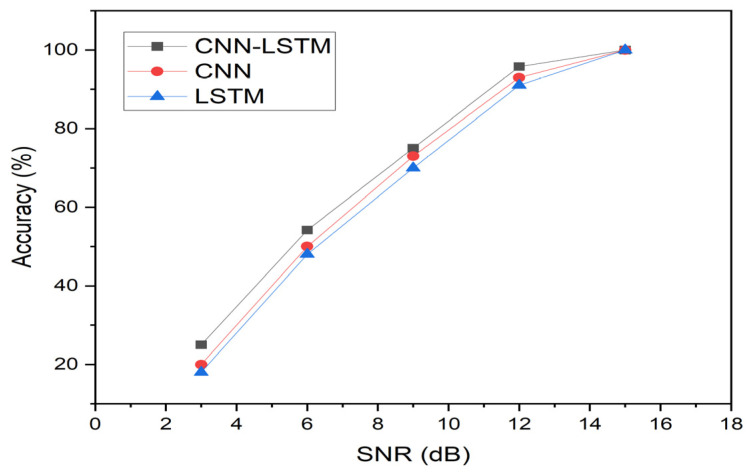
Accuracy of the models for a pipe embedded in concrete under different noise levels.

Figure 16 shows the ability of the CNN-LSTM model to detect multiple defects, as compared to those of the CNN and LSTM approaches. Clearly, with the increase of the noise level, all deep learning approaches exhibited a significant drop in their accuracy, suggesting that signals under a concrete embedment are sensitive to noise interference, as compared to the signals under a soil medium, as discussed in Section 4. As a comparison, the CNN-LSTM model could outperform CNN and LSTM in all cases, as observed in Section 4.

Specifically, when the noise level was 15 dB, the CNN-LSTM model maintained a high accuracy of 100%, and the AUC value was 1.0. However, with the increase of the noise level, the accuracy of the prediction by the CNN-LSTM model dropped dramatically, particularly when the noise level was 3 dB, and the accuracy of the CNN-LSTM model was far less than 50%, that is, the noise level totally misled the data classification. Both the CNN and the LSTM approaches received an identical impact from the noise and performed even worse when the noise level increased.

## 6. Conclusions

This study provides a comprehensive analysis of deep-learning-based signal processing of ultrasonic guided waves and damage detection for metallic pipelines via the CNN-LSTM hybrid model. Twenty-nine features, including time, frequency, and time–frequency domains, were determined to evaluate the data classification. Six types of mechanical defects in pipe structures were designed to demonstrate the effectiveness of the proposed method. As a comparison, the CNN and LSTM models were selected. To further evaluate the robustness of the signal processing and model training, noise interference on the signal processing was investigated. The main findings could be summarized as follows:

- The results revealed that the CNN-LSTM hybrid model exhibited a higher accuracy for decoding signals of ultrasonic guided waves for damage detection, as compared to individual deep learning approaches (CNN and LSTM), particularly under high noise interference.
- The results also confirmed that predetermined features, including time, frequency, and timey-frequency domains, improved the data classification. Interestingly, while it is well known that deep learning approaches could outperform shallow learning ones that often require hand-crafted features and, thus, could provide high capability for data classification through end-to-end manner with fewer physics restraints (“black box”), the election of features with certain physics (“physics-informed” feature extraction) could significantly improve the robustness of deep learning approaches.
- The data reduction (PCA and KPCA) used for the deep learning training/testing networks in this study display no apparent improvement to the data classification. However, with the increased volume of datasets, these methods could improve the efficiency in terms of shortening the computation time.
- The accuracy of the deep learning approaches could be dramatically affected by noise, which could stem from measurement and environment. The CNN-LSTM model still exhibited a high performance when the noise level was relatively low (e.g., SNR = 9 or higher), but the prediction dropped gradually to an unacceptable limit when the noise level in relation to SNR was 6, with the amplitude of the noise level approaching to that of the signals themselves. In comparison, the CNN and LSTM models failed early as expected, when the noise level was much higher.
- Although this study attempted to provide a comparison to understand the effectiveness of the hybrid deep learning model, there are still certain drawbacks that could be improved in the future. The first one is the dataset which was limited to six common defects and may not be able to account for broader applications. The simple case we chose to try to demonstrate the concept may not account for more complicated signal propagation, reflection, and scatters, which could challenge the effectiveness of the proposed method.

## Figures and Tables

**Table 1:** Time-domain characteristic indicators.

Dimensional Time Domain (with 10 Indicators)			
Feature Index	Expressions	Features Index	Expressions
Mean value	$\bar{X}=\frac{1}{N}\sum_{i=1}^{N}x_{i}$	Kurtosis	$\beta =\frac{1}{N}\sum_{i=1}^{N}x_{i}^{4}$
Root-mean-square value	$X_{rms}=\sqrt{\frac{1}{N}\sum_{i=1}^{N}x_{i}^{2}}$	variance	$\sigma_{x}^{2}=\frac{1}{N-1}\sum_{i=1}^{N}{\left(x_{i}-\bar{X}\right)}^{2}$
Square-root amplitude	$X_{r}={\left[\frac{1}{N}\sum_{i=1}^{N}\sqrt{\left|x_{i}\right|}\right]}^{2}$	maximum value	$X_{max}=max\left\{\left|x_{i}\right|\right\}$
Absolute mean amplitude	$\left|\bar{X}\right|=\frac{1}{N}\sum_{i=1}^{N}\left|x_{i}\right|$	minimum value	$X_{min}=min\left\{x_{i}\right\}$
Skewness	$\propto =\frac{1}{N}\sum_{i=1}^{N}x_{i}^{3}$	peak-to-peak value	$X_{p-p}=\max\left(x_{i}\right)-\min(x_{i})$
Dimensionless time domain (with 6 indicators)			
Waveform Index	$S_{f}=\frac{X_{rms}}{\left|\bar{X}\right|}$	peak index	$C_{f}=\frac{X_{max}}{X_{rms}}$
pulse index	$I_{f}=\frac{X_{max}}{\left|\bar{X}\right|}$	margin index	${CL}_{f}=\frac{X_{max}}{X_{r}}$
kurtosis index	$K_{v}=\frac{\beta }{x_{rms}^{4}}$	Skewness Index	$S=\frac{\propto }{x_{rms}^{3}}$

**Table 2:** Frequency-domain characteristic indicators (with 13 indicators).

Number	Expressions	Number	Expressions
1	$p_{1}=\frac{\sum_{k=1}^{K}s(k)}{K}$	8	$p_{8}=\sqrt{\frac{\sum_{k=1}^{K}{f_{k}}^{4}s(k)}{\sum_{k=1}^{K}{f_{k}}^{2}s(k)}}$
2	$p_{2}=\frac{\sum_{k=1}^{K}{\left(s\left(k\right)-p_{1}\right)}^{2}}{K}$	9	$p_{9}=\frac{\sum_{k=1}^{K}{f_{k}}^{2}s(k)}{\sqrt{\sum_{k=1}^{K}s(k)\sum_{k=1}^{K}{f_{k}}^{4}s(k)}}$
3	$p_{3}=\frac{\sum_{k=1}^{K}{\left(s\left(k\right)-p_{1}\right)}^{3}}{K{\left(\sqrt{p_{2}}\right)}^{3}}$	10	$p_{10}=\frac{p_{6}}{p_{5}}$
4	$p_{4}=\frac{\sum_{k=1}^{K}{\left(s\left(k\right)-p_{1}\right)}^{4}}{Kp_{2}^{2}}$	11	$p_{11}=\frac{\sum_{k=1}^{K}{\left(f_{k}-p_{5}\right)}^{3}s\left(k\right)}{Kp_{6}^{2}}$
5	$p_{5}=\frac{\sum_{k=1}^{K}f_{k}s(k)}{\sum_{k=1}^{K}s(k)}$	12	$p_{12}=\frac{\sum_{k=1}^{K}{\left(f_{k}-p_{5}\right)}^{4}s\left(k\right)}{Kp_{6}^{4}}$
6	$p_{6}=\sqrt{\frac{\sum_{k=1}^{K}{\left(f_{k}-p_{5}\right)}^{2}s(k)}{K}}$	13	$p_{13}=\frac{\sum_{k=1}^{K}{\left(f_{k}-p_{5}\right)}^{0.5}s\left(k\right)}{Kp_{6}}$
7	$p_{7}=\sqrt{\frac{\sum_{k=1}^{K}{f_{k}}^{2}s(k)}{\sum_{k=1}^{K}s(k)}}$		

**Table 3:** Confusion matrix for binary classification.

		Predicted	
		Negative	Positive
Actual	Negative	TN	FP
	Positive	FN	TP

**Table 4:** Data label and damage type.

Sample ID	Damage Type	Training Sample	Testing Sample
P-1	pipe with a small notch located at 1/3 L away from the left side	240	96
P-2	pipe with a big notch located at 1/3 L away from the left side and a weldment at 2/3 L away from the left side		
P-3	pipe with a small notch at 1/3 L and a weldment at 2/3 L away from the left side		
P-4	pipe with a big notch shaped damage		
P-5	pipe with epoxy coating without damage		
P-6	pipe with epoxy coating with a weldment at 2/3 L away from the left side.		

**Table 5:** The classification accuracy for different deep leaning models.

Deep Learning Models	Input	Output (Accuracy)	Training Time (s)
CNN	twenty-nine feature parameter series	85.4%	30
LSTM		86.5%	37
CNN-LSTM		94.8%	45

**Table 6:** Accuracy and AUC for the CNN-LSTM hybrid model with different input data.

Deep Learning Models	Input	Accuracy	AUC
CNN-LSTM	With Original data	77.1%	0.770
	With Denoised data	87.5%	0.855
	With twenty-nine feature parameter series	94.8%	0.950
	Twenty-nine feature parameter series with PCA	93.8%	0.935
	Twenty-nine feature parameter series with KPCA	92.7%	0.930

**Table 7:** The classification accuracy of the three models for different SNR.

Input	SNR (dB)	Accuracy		
		CNN	LSTM	CNN-LSTM
Twenty-nine feature parameter series (original signal)	NAN	85.4%	86.5%	94.8%
Twenty-nine feature parameter series (noised signals)	3	25.0%	28.8%	33.3%
	6	65.5%	67.7%	75.0%
	9	76.8%	78.5%	83.3%
	12	80.0%	83.0%	85.4%
	15	83.0%	84.6%	93.8%

**Table 8:** The AUC values of the three models at different noise levels.

Input	SNR (dB)	AUC		
		CNN	LSTM	CNN-LSTM
Twenty-nine feature parameter series (original signal)	NAN	0.850	0.855	0.950
Twenty-nine feature parameter series (noised signals)	3	0.250	0.280	0.335
	6	0.655	0.700	0.720
	9	0.775	0.780	0.840
	12	0.800	0.830	0.855
	15	0.830	0.845	0.950

## Data Availability

Data will be available based on request.
